# The effects of flooding on mental health: Outcomes and recommendations from a review of the literature

**DOI:** 10.1371/4f9f1fa9c3cae

**Published:** 2012-05-30

**Authors:** Carla Stanke, Virginia Murray, Richard Amlôt, Jo Nurse, Richard Williams

**Affiliations:** Environmental Public Health Scientist, Extreme Events and Health Protection Section, Health Protection Agency, London UK; Head of Extreme Events and Health Protection, Health Protection Agency, London UK; Scientific Programme Leader - Behavioural Science, Emergency Response Department, Health Protection Agency; Senior Advisor, Public Health Services, World Health Organisation, Regional Office for Europe; Professor of Mental Health Strategy, Welsh Institute for Health and Social Care, University of Glamorgan, and Consultant Child and Adolescent Psychiatrist, Aneurin Bevan Health Board, UK

## Abstract

Introduction While most people who are involved in disasters recover with the support of their families, friends and colleagues, the effects on some people’s health, relationships and welfare can be extensive and sustained. Flooding can pose substantial social and mental health problems that may continue over extended periods of time. Flooding can challenge the psychosocial resilience of the hardiest of people who are affected. Methods The Health Protection Agency (HPA) undertook a review of the literature published from 2004 to 2010. It is intended to: assess and appraise the epidemiological evidence on flooding and mental health; assess the existing guidance on emergency planning for the impacts of flooding on psychosocial and mental health needs; provide a detailed report for policymakers and services on practical methods to reduce the impacts of flooding on the mental health of affected people; and identify where research can support future evidence-based guidance. The HPA identified 48 papers which met its criteria. The team also reviewed and discussed relevant government and non-government guidance documents. This paper presents a summary of the outcomes and recommendations from this review of the literature. Results The review indicates that flooding affects people of all ages, can exacerbate or provoke mental health problems, and highlights the importance of secondary stressors in prolonging the psychosocial impacts of flooding. The distressing experiences that the majority of people experience transiently or for longer periods after disasters can be difficult to distinguish from symptoms of common mental disorders. This emphasises the need to reduce the impact of primary and secondary stressors on people affected by flooding and the importance of narrative approaches to differentiate distress from mental disorder. Much of the literature focuses on post-traumatic stress disorder; diagnosable depressive and anxiety disorders and substance misuse are under-represented in the published data. Most people’s psychosocial needs are met through their close relationships with their families, friends and communities; smaller proportions of people are likely to require specialised mental healthcare. Finally, there are a number of methodological challenges that arise when conducting research and when analysing and comparing data on the psychosocial and mental health impacts of floods. Conclusions The HPA’s findings showed that a multi-sector approach that involves communities as well as agencies is the best way to promote wellbeing and recovery. Agreeing and using internationally understood definitions of and the thresholds that separate distress, mental health and mental ill health would improve the process of assessing, analysing and comparing research findings. Further research is needed on the longitudinal effects of flooding on people’s mental health, the effects of successive flooding on populations, and the effects of flooding on the mental health of children, young people and older people and people who respond to the needs of other persons in the aftermath of disasters. Corresponding author: Carla Stanke Address: Health Protection Agency 151 Buckingham Palace Road London SW1W 9SZ E-mail: carla.stanke@hpa.org.uk Fax: 020 7811 7759 Telephone: 020 7811 7161

## Introduction

Flooding is now the most frequent type of major disaster. Over the last 10 years, floods in Europe have killed more than 1,000 people and affected over 3.4 million others^1^. In common with other types of major incidents or disasters, the effects on people’s health, relationships and welfare can be extensive. However, flooding also stands out from some other types of disasters because, often, it is possible to prevent flooding by using flood-prevention measures, and because of the lengthy recovery period afterwards, which increases the risks of secondary stressors arising and the impact of worry about recurrence on people who are affected by floods.

Furthermore, the impacts of climate change are likely to increase the occurrence of flooding. Projected climate-related increases in precipitation are likely to make floods more frequent and severe. Coastal flooding is likely to threaten up to 1.6 million more people every year in the European Union^2^.

Accounts of the psychosocial impacts of flood events suggest that they can have significant effects on people’s wellbeing, relationships and mental health. Flooding can pose substantial social and welfare problems that may continue over extended periods of time because of not only being flooded (the primary stressor), but also because of the secondary stressors (those stressors that are indirectly related to the initial extreme event, i.e., economic stress associated with re-building) that arise as people try to recover their lives, property and relationships. Flooding can challenge the psychosocial resilience of the hardiest of people who are affected.

In their 2005 review on the global health impacts of flooding, Ahern et al^3^ report a number of epidemiological studies which examined the effects of flooding on common mental disorders (including anxiety and depression), post-traumatic stress disorder (PTSD) and suicide. Most studies exploring the effects of flooding on common mental disorders came from high or middle-income countries, and results revealed significant increases in depression, anxiety and psychological distress among flooded adults; relatively few studies examined the effects of flooding on children, but those that did revealed increases in aggression, bedwetting and moderate to severe stress symptoms. Studies showing increases in PTSD following flooding came from Europe and North America, with limited evidence reported about suicide in relation to flooding.

This paper provides a summary of the outcomes and recommendations from a recent literature review entitled *The effects of flooding on mental health* 4. Access to the full report can be gained at:  http://www.hpa.org.uk/webw/HPAweb&HPAwebStandard/HPAweb_C/1317131788841

Importantly, this paper offers a short version of the critique that appears in the HPA’s report concerning recent peer-reviewed literature and authoritative guidance for delivering responses to peoples’ needs for psychosocial and mental health care. It also offers an overview of the HPA’s recommendations to the responsible authorities and agencies about developments to the research agenda and service developments that are informed by the messages from recent research and expert opinion.

In 2009-2010, the Department of Health for England asked the HPA to contribute to its *New Horizons: A shared vision for mental health policy* 5. The New Horizons policy states that mental health problems are common as are mental disorders that spring from environmental as well as physical and social risks. Since then, a new government has been elected in the UK and health policy for public mental health matters has been taken forward by the strategy titled No Health Without Mental Health^6^. That policy sustains the general approach in New Horizons and affords prominence to developing people’s resilience and preventing people from developing mental disorders. One of the environmental risks that may threaten people’s mental health is posed by disasters, which include flooding. The HPA responded by:

- Assessing and appraising the epidemiological evidence on flooding and mental health;

- Assessing the existing guidance on emergency planning for the mental health needs of people who are affected by flooding;

- Providing a more detailed report for policymakers and services, which highlights the evidence about the impact of flooding on people’s mental health, and which provides a summary of such information as is available about practical methods to reduce these impacts through the work of public sector services and by their collaboration with other non-governmental agencies; and

- Identifying where and how research can support development of future evidence-based guidance.

This paper summarises the methodology used in that review, and sets the findings into the context framed by: current and immediate past policy in England; guidance offered by international governing bodies (the World Health Organization and NATO) and non-governmental authorities; and a selection of recent advice from academic and other sources. The discussion identifies the challenges faced by the researchers in this area. It also draws together key themes and recommendations that emerge in the HPA’s report.

## Methods

Two main types of source were used to examine the effects of flooding on people’s mental health. First, a review of the published academic literature and, second, guidance from governmental, non-governmental and other authoritative sources and selected papers that describe how services might be mounted in response to people’s psychosocial and mental health needs after flooding.

THE OBJECTIVES OF THE LITERATURE REVIEW

The specific objectives of the literature review were:

- To review the epidemiological evidence about the mental health impacts on people who have been affected by flooding by critically appraising published studies of floods that have occurred world-wide; and

- To identify gaps in current knowledge about reducing the psychosocial and mental health impacts of flooding, its effects on public mental health, and people’s needs for mental healthcare.

LITERATURE SEARCH STRATEGY

The authors collected evidence through a systematic review of the literature that was published between 2004 and 2010. The methodology used was similar to that used in a global epidemiological review that was published in 2005 on the health impacts of flooding^3^. The 2005 epidemiological review included a section on the mental health effects of flooding; searching the literature from 2004 onwards allowed the authors to review the results of more recent studies which were not included in the epidemiological review published in 2005, building upon what was already established with new studies. The authors developed an algorithm and used the Scopus search engine to search for all papers with the specified search terms in their titles, abstracts or as keywords (Scopus is a search tool that scans and has access to many large academic databases including Medline (pubmed), Embase, Psychinfo, and covers 15,000 peer-reviewed journals as well as conference proceedings and book series).

INCLUSION CRITERIA

The inclusion criteria were that the epidemiological papers selected should have been published in peer-reviewed journals in the subject areas of: medicine, nursing, psychology, pharmacology and toxicology. The authors also sought data that is available from government and non-governmental organisations and took advice from experts in the field on what research was in press or in process as well as seeking their knowledge of what other sources of information they knew.

EXCLUSION CRITERIA

The authors excluded papers if they were:

- Irrelevant to the search such as use of the words ‘flood’ and ‘flooding’ to mean inundation; biochemistry, genetics and molecular biology;

- Conference papers, dissertations, older papers, editorials, commentaries, meta-analyses and unpublished manuscripts; or

- Not published in English, and not published between 2004 and 2010.

The authors manually reviewed references from full text papers in order to identify additional articles that met the inclusion criteria. In addition, the authors searched the databases listed here in order to identify other recent epidemiological reviews:

- DARE (Database of Abstracts of Reviews of Effects);

- CDSR (Cochrane Database of Systematic Reviews);

- EPPI (Evidence for Policy and Practice Information Centre); and

- DoPHER (Database of providing health effectiveness reviews).

The search generated 3,585 references and this number was reduced to 827 papers when the authors applied their exclusion criteria. The authors assessed all of the papers for their suitability for inclusion in the review by applying the strict epidemiological criteria that are cited in Ahern et al^3^ .

THE PAPERS REVIEWED

The HPA found 48 papers on mental health that fit the strict criteria. The authors included all of them in the review, but gave greatest weight to studies that were based on epidemiological designs with controlled comparisons.

Table 1 summarises the epidemiological papers that are assessed in the HPA’s report (reference numbers in parenthesis).

**Table 1: Distribution of research papers by flood event (reference numbers in parentheses)**

**Table:** 

Flood event	Number of papers
American Hurricanes 2004/5 (7-34)	28
Poland 1997 (35;36)	2
Mexico 1999 (37;38)	2
China, Hunan province 1998 (39-41)	3
Vietnam, Xangsane Typhoon 2006 (42)	1
Sri Lanka, Tsunami 2004 (43)	1
Germany 2002 (44)	1
Korea 2006 (45)	1
Carlisle, UK 2005 (46)	1
Iowa, US 1993 (47;48)	2
Mississippi River (Illinois and Missouri)1993 (49;50)	2
Italy 1996 (51)	1
UK 2007 (52;53)	2
UK 1998-2002 (54)	1
Total	48

Twenty-eight (28) of the papers assessed people’s mental health after hurricanes in America and 20 after floods. The authors made the decision to include hurricanes leading to flooding because of the difficulties in classifying disasters. We concluded that it was important to include the extensive data on the mental health of people affected by hurricanes because of the health impacts of the floods immediately following Hurricane Katrina.

The majority of papers that the authors reviewed consider population samples of adults who are affected by a particular flood. A number of papers report research on samples taken from specific populations: six relate to people aged under 18 9 12 13 16 40 41; two studies were conducted in evacuation shelters after Hurricane Katrina 17 18; one specifically sampled ethnic minorities 15; one assessed patients evacuated from a heart centre^44^; and one study was of pregnant women 14. The majority of the studies were cross-sectional.

The timing of the studies, relative to the flood considered by the research, varied from data collected pre-event, to data collected after zero to two weeks, and up to eight years after flooding. However, the vast majority of the studies took place between six months and 24 months after the index event. The size of the population samples was also very diverse, ranging from 50 to 33,000 people.

THE REVIEW OF RECENT GUIDANCE DOCUMENTS

The authors reviewed recent government policy in England and other governmental and non-governmental guidance documents of international standing as well as relevant papers which offer theoretical standpoints^55^ ^56^ ^57^ ^58^ ^59^ ^60^ ^61^ ^62^ ^63^ ^64^. The intention was to identify documents in the public domain that outline, directly or by implication, models of care for responding to the psychosocial and mental health impacts of disasters in general and, if possible, flooding in particular. In this regard, we were greatly assisted by advice from experts.

As a result, the authors analysed the content of guidance documents, research reports, and other papers. They include the current policy on psychosocial and mental health care in the set of contemporary emergency preparedness guidance published by the Department of Health for England^59^ and a report on the research conducted by the University of Lancaster on the psychosocial and welfare impacts of the floods that occurred in England in 2007^63^. Each of the documents that the HPA reviewed offers practical guidance for policymakers, strategic and operational managers, public health services and clinical services. Importantly, their advice fits well with the evidence that emerged from the literature review and which is reviewed in the HPA’s report.

## Results

CORE FINDINGS FROM THE PAPERS REVIEWED IN THE REPORT

The studies analysed in the report are clear that flooding is very stressful and that the stress continues for a long time after the water has receded. Flooding affects people of all ages and it can herald: bereavement; economic problems for families; behavioural problems in children; increased substance use and/or misuse; increased domestic violence; as well as exacerbating, precipitating or provoking people’s existing problems with their mental health.

Often, people’s experiences, which reflect the personal and social meanings of the event for them, and the understandings and meanings they derive from it, have more influence on the psychosocial impact of the event than the event itself. Recovery from distress after disasters, including flooding, is characterised by adaptation to circumstances that have changed and by rebuilding communities.

Many people experience distress that may be relatively transient after any disaster and being distressed temporarily is not antithetical to people also being resilient. Furthermore, the wider literature shows that the experiences of people who are distressed in the aftermath of all disasters including floods, are not always easy to distinguish from the symptoms of common mental disorders. On the other hand, the research suggests that the incidence and prevalence of common mental disorders after flooding is substantially increased and that these disorders can persist long after the flooding has passed. This stresses the importance of planning for and providing effective and timely public mental health and clinical responses.

The threshold between what might be considered a common or anticipated response to an extreme event and what is indicative of a person developing a disorder are difficult to define. Much turns on the severity, duration, impacts of these experiences on people’s lives, and, particularly, the trajectory of their reactions over time, and the severity and persistence of any dysfunction they accrue, when it comes to differentiating distress and disorder. The authors found that the focus in the literature is on post-traumatic stress disorder (PTSD). While that is valuable, it is also accompanied by a relative neglect of the crucial wider background morbidity that is found in all populations, including after disasters. For example, depression is a diagnosis that is under-represented in the published data. However, a consistent finding across many studies is that people’s level of exposure to the event and their earlier exposures to other traumatic experiences are strongly associated with PTSD.

When considering PTSD, findings reveal that the symptoms may not decline over time as quickly as was thought previously. The authors found, though, that social cohesion has a significant effect on susceptibility to symptoms of PTSD and it, therefore, must be considered when developing public health strategies.

As regards people who develop mental disorders, risk factors and co-variants did not a have a constant association with poorer mental health across all the studies, partly due to methodological differences and partly because of the unique characteristics of each flood. However, as in general population studies, levels of exposure to the event(s), gender, age, and socio-economic status were generally associated with mental ill health.

There is a lack of studies which have investigated the impact of flooding on the mental health of children, young people and older people. There are, however, indications that both children and older people suffer PTSD after flooding and that the prevalence figures may well be greater than those that are found for adults of working age. Children, young people and older people may be more vulnerable than are adults of working age because they are dependent on adults’ responses to the floods that affect families. Parents’ wellbeing, for example, affects the quality of their parenting; people’s direct experiences and those that affect their carers may, separately and in interaction, either protect them or intensify the negative effects on children and older people.

As the review observes, the studies analysed in it illustrate how floods can have great impacts on people’s psychosocial needs and mental health. Evidence-based guidance on the factors that could influence the course of an illness are valuable when developing tools to minimise the psychosocial and mental health impacts of flooding. As an example, the extended timeframe of the impacts of flooding on people, their homes and their communities are such that the effects of secondary stressors are highly important because they prolong the welfare, physical and psychosocial impacts. Recognition of the longer timeframe in which adequate welfare, psychosocial and mental healthcare responses are required is an important lesson that has been learned from floods in the past, and that lesson is supported strongly by the findings from the review.

PLANNING, DESIGNING AND DELIVERING HEALTHCARE RESPONSES

Documents that set out current policy in planning, designing and delivering healthcare responses for people who experience flooding and general disasters were reviewed^4^ ^9^ ^59^ ^65^. In addition, other guidance^55^ ^56^ ^57^ ^58^ ^60^ ^61^ ^62^ ^64^ and research^63^ which offers theoretical standpoints and outline, directly or by implication, models of care for responding to the psychosocial and mental health impacts of disasters were also reviewed. The documents offer access to practical guidance for managers, public health services and clinical services. The key themes that emerge from this guidance include:

- The importance of adopting a multi-sector approach to promoting wellbeing and recovery that involves communities as well as agencies;
- Most people who are affected by flooding are remarkably resilient;
- Many people who are affected face psychosocial challenges and most have distressing experiences for which they require psychosocial support; these responses can be anticipated, as should be their needs for support;
- Most people’s psychosocial needs are met by people who are close to them, but some people may require more substantial psychosocial care and approaches that are based on the principles of psychological first aid are appropriate;
- A substantial minority of people who are affected by flooding are at risk of developing a mental disorder and they may require healthcare services that include psychosocial care and mental healthcare;
- The Strategic Stepped Model of Care^59^ ^64^ is a very useful tool.

The Strategic Stepped Model of Care allows planners to take into account sources of personal and collective social support, and how responses to events are developed. It also allows psychosocial care and mental healthcare to be planned and delivered in integrated ways that follow the trajectories of the needs of people who require them. Thereby, the responsible authorities can create a dynamic, flexible and needs-led approach to mounting effective responses to flooding. Understanding the overlaps between the steps can be facilitated by incorporating knowledge of the:

- Nature of each flood and its consequences;
- Primary and secondary stressors; and
- Means of delivering care.

People’s psychosocial needs, and the mental disorders that they might develop as a consequence of their being flooded, pose core challenges for community, public health, primary care and specialist mental healthcare services.

Public health measures that are put in place after floods should include considering the social as well as psychological impacts of events, and especially because evidence suggests that good social support can act as a protective factor against negative psychological and psychiatric impacts of being flooded. Furthermore, recently published results from a randomized trial provide provisional evidence that psychosocial interventions, which target the resilience resources (e.g. emotional engagement and social connectedness) of veterans who have PTSD, may alleviate their anxiety and depressive symptoms and improve their positive emotional and cognitive functioning^66^.

After being affected by floods, people may have substantial psychosocial needs, and/or develop mental health problems and mental disorders in the short-, medium- or long-terms. People who are affected need responding organisations to: support their psychosocial resilience and maintain their emotional wellbeing; recognise and respond to their distress; and take actions to prevent the onset of additional mental health problems or disorders. These responses should be flexible and varied according to people’s needs and according to their sources of social support, as well as their economic and social circumstances. Understanding flooding in these terms should aid and direct the responses from services as well as from communities. This applies not only to initial emergency responses, but also to support and reconstruction during recovery as many people and communities can experience continuing social and economic disruption after flooding. A multi-sector approach that involves communities and families as well as agencies is the best way to achieve these effects. It is important to recognise that family and community assets are vitally important to maintaining and promoting personal and collective psychosocial resilience.

Everyone is likely to require continuing psychosocial support. Most people’s psychosocial needs are met through their close relationships with their families, friends and communities. However, some people require assessment by primary care services if their symptoms persist or are associated with dysfunction. A smaller proportion of people is likely to require referral for specialised mental healthcare.

These findings support the requirement for a public mental health approach to flooding that comprises both universal and targeted plans and interventions, which are well coordinated with adequate, timely access for people in need as well as long-term availability of specialist care for a sizeable minority of affected people. Primary care and specialist mental health services should recognise the long duration of the stress that affects people after they are inundated, the high frequency of secondary stressors and the author’s finding that PTSD symptoms do not necessarily remit rapidly after flooding.

## Discussion

KEY THEMES

Core findings and recommendations that emerged from the guidance inform the themes that follow.

1. It is important to understand stress, and the stressors that are inflicted upon people by floods and how they cause short-term distress in many people, influence their medium- and longer-term wellbeing, and affect the mental health of persons and populations.
2. Primary stressors are inherent in all disasters and encompass any experiences that people have that are directly related to, or consequent on their exposure to disasters.
3. A majority of people experience distress after disasters. But, personal and collective psychosocial resilience are inherent in each population, and families, communities and non-statutory and statutory services offer protection for people against psychosocial adversity during and after extreme events.
4. Secondary stressors follow on from, or are consequential on primary stressors: they include infrastructure failure and challenges to people, families and communities returning to normality and repairing buildings, or failure to adjust to the ‘new normality,’ i.e., the new set of living circumstances, that ensues after disasters.
5. People’s psychosocial experiences can be shaped by the origin and delivery of care and the timeframes of activation of stressors: this makes it possible to describe how and where public health responses should be delivered.
6. People’s psychosocial experiences in the aftermath can oscillate between distress and recovery, and the aftermath of their recovery from floods tends to be a phase of, at least, medium-term duration, which can endure for weeks, months or years. This is because:

- Secondary stressors often arise during the responses to the clean up, recovery and rebuilding phases after flooding.
- Some people’s experiences may be of the quantity, severity and duration or are associated with sustained dysfunction such that it is appropriate to call them symptoms of mental disorders.
- Support networks may not provide enough support for a small proportion of people, or some people may not recover from distress even though stressors are removed. In this situation, more persistent problems, or mental disorders, can develop, or pre-existing ones are provoked.

People who are affected in these ways should be assessed by the primary care services and some of them require treatment by the specialist mental health services.

COMPLEXITIES AND LIMITATIONS

It became apparent to the authors that there are a substantial number of methodological complexities and challenges which emerged in their review of the literature. They include:

- The lack of universally agreed definitions used when researching disasters. The authors found that researchers may use the same and different terms to describe people’s experiences and responses, including inconsistencies in their use of the different classifications of mental disorders, which have differing thresholds for caseness;
- The wide variety of methodologies that are used across the studies that were scrutinised;
- The broad range of mental disorders that are described and assessed in the literature;
- Diversity in the co-variants that different researchers have assessed;
- The use of a variety of different diagnostic measurement tools; and
- Complexity when classifying the nature of each flood and population that was exposed to it.

In addition to these inconsistencies, the recent literature on disasters, especially that on flooding, has tended to focus on the single and narrow concept of PTSD. The result is that, first, less research has been conducted on the psychosocial needs of people who are distressed rather than disordered. Second, the canon of research has tended to neglect the crucial wider and, sometimes, more prevalent morbidity that is found in all populations, including that which affects people who are involved in flooding.

This is why much of the evidence relates to people who develop a narrow selection of mental disorders. There are two other groups of people about which healthcare services need to know more: people who develop disorders other than PTSD, which the literature shows is very often co-morbid with other mental disorders; and the wider group of people who are distressed by events, temporarily or otherwise (a highly relevant matter after flooding) and who have psychosocial needs that relate to sustaining their mental health and emotional wellbeing and also preventing their developing mental disorders.

THE WAY FORWARD FOR RESEARCH

The authors identify significant research gaps, which, if filled, could support better design and delivery of future psychosocial and public mental health responses, and improved primary and secondary mental healthcare responses to people’s needs before and after flooding.

In general terms, more research is required, which studies:

- The responses of, and impacts on populations before and after untoward and extreme events, major incidents and disasters including flooding;
- The impacts of major incidents and disasters, including flooding, on people’s psychosocial experiences in the short-, medium- and longer-terms;
- The impacts of successive floods on populations;
- The contextual and subjective, qualitative features of peoples’ experiences which distinguish distress after disasters from the symptoms of mental disorders;
- The longitudinal effects of major incidents and disasters, including floods, on people’s mental health and ill health. Better use could be made of national psychiatric morbidity survey programmes, which could provide useful baseline data for populations that are flooded subsequently, as well as comparison/control data from populations which reside in non-flooded areas;
- Co-morbidity of mental disorders and how this can affect treatment plans: each disorder is assessed discretely in most of the papers reviewed and this does not necessarily reflect the reality of the experiences of people who are affected. When specific plans or protocols are developed for intervening with people who have particular disorders, they should reflect real situations in which co-morbidity is an important matter for treatment algorithms;
- The consequences and implications of diagnosing PTSD after people are flooded; and
- The mechanisms for people developing, and the consequences of them having, PTSD, including their rates and trajectories of recovery.

The methodological variables and limitations that the HPA identifies in its report point to the importance of improving the quality of research. Further research should focus on:

- Using overt definitions of psychosocial need, mental health and mental ill health that are agreed, understood and used internationally and which include overt thresholds for caseness;
- Achieve development of systems for cross comparison of research findings; and
- Take forward findings to formal meta-analysis to identify better welfare and public health guidance and professional practice.

Better design of research instruments would help researchers to appraise people’s common experiences and symptoms rather than a narrow subset, and might provide better information about the duration, severity and effects of people’s experiences and symptoms. Subsequently, it will be possible to look at the impacts on public health of people’s psychosocial experiences and needs as well as the effects on populations of mental disorders.

We identify the requirement for more research on: children and young people; older people; and people who respond to others needs in the aftermath of major events or disasters. This would help the research community to address: who or which groups of people are more at risk; how and why certain groups of people suffer more; and what should be done in addition to current interventions to respond effectively to people’s needs.

The authors did not explore in their report the secondary health impacts of disasters, the pathways from disasters to mental ill health, or the consequential impacts of developing a mental disorder. The authors considered one study that researched the somatic effects of mental ill health^9^, another that researched substance misuse^50^, and others that have considered gender-based violence^27^ ^28^ ^29^. They conclude that clarification is required about what constitutes best practice in each of these areas.

Further work on the effects of flooding on mental health could follow the Preferred Reporting Items for Systematic Reviews and Meta-Analyses (PRISMA) statement^67^.

RECOMMENDATIONS

It is now clear that psychosocial distress and mental disorders may be provoked or exacerbated by people’s exposure to floods.

Together, the peer-reviewed research papers and guidance reported by the HPA allow the authors of that report to make recommendations for a variety of public agencies. They are presented here in general terms so that they can be interpreted and applied in all countries and jurisdictions.

Policymakers

It is clear that there is sufficient evidence available to show that the short- and long-term psychosocial and mental health consequences of flooding should be a high priority for policymakers. Development of evidence-based policy in this area should be a priority for people who are responsible for planning for and responding to emergencies, healthcare, social care, and mental healthcare services. Government departments should work together to achieve integrated policies in order to avoid ‘recovery gaps,’ i.e. gaps in societal responses that arise in the period after the emergency responses have ended when people must rely on the services that are available ordinarily in communities to support their continuing recovery^63^.

Government organisations are advised to work with research funding agencies to identify priorities and to develop research evidence of increasing quality and relevance to policymakers. In particular, they should be aware of the HPA’s commentary on the existing research in this topic area and of its recommendations for future research and how it is conducted.

Emergency Planners

People who have responsibilities for emergency preparedness within governmental, non-governmental, service planning, and delivery organisations, and within the commercial sector, should also consider the short- and long-term psychosocial and mental health consequences of flooding. They should work with the agencies that are responsible for managing the environments in which people live to achieve these aims because measures for preventing floods can reduce the health impacts on populations.

Social Care Services

The concept of psychosocial resilience, with its personal and collective components, points to the importance of mitigating the distressing effects as well as the social and health risks posed by flooding. Social cohesion of communities and families before calamities occur and their restoration as soon as possible afterwards, restoring communications between people, and keeping families together are core to reducing people’s suffering and promoting their effective recovery. These actions are important components of Psychological First Aid^68^, which is now agreed internationally to be an important set of principles for psychosocial care.

Agencies that are responsible for social care and commercial companies, including builders and insurance companies that are involved in rebuilding and restoration, should work together to ensure that there are no ‘recovery gaps’^63^.

Healthcare Services

All healthcare agencies should be aware of the distress that flooding may cause people who are affected. The literature is not clear about the impact of floods in provoking more people than might otherwise do so to develop anxiety disorders, depression and PTSD. This is because of limited availability of population comparisons, and before and after evaluation studies. Local healthcare services should be aware that a substantial minority of people may develop mental disorders in the medium- and longer-terms after flooding. Also, whatever the relative contribution of flooding as a causative agent, floods are likely to bring people’s pre-existing disorders to presentation.

This health intelligence should inform the preparedness of healthcare agencies and their practical plans. Specialist mental healthcare agencies should be prepared to provide advice to emergency planners, community healthcare services, and social care organisations. They should also be available to deliver focused specialist services for people who may have mental disorders that are provoked or exacerbated by their involvement in floods.

## Conclusions

The frequency of floods is increasing. The mortality relating to flooding is variable and depends on the enormity or otherwise of each extreme event and the capability of the rescue and recovery services. But, when compared with other types of disaster, the mortality may be interpreted as low when floods occur in well-prepared areas of the world. However, this should not blind policymakers, service designers and practitioners to the substantial morbidity that is posed by flooding.

The Health Protection Agency’s report on the outcomes and recommendations from the peer-reviewed literature, and selected governmental policy and non-governmental guidance, provides a picture that is based on key messages from research and expert consensus. It identifies clearly the requirement for more research that is of improved clarity of purpose and method. The need for greater discipline in defining and using terminology is another important message. The authors identify the implications of their review for policymakers, the responsible government agencies, and health and social care services.

The HPA has identified the responsibilities that should fall on a number of public and other organisations to plan and deliver improved services to support people who are affected by flooding with the intention of reducing their suffering, reducing the risks of them developing long-term mental disorders that are so very costly across the full diversity of meaning of that concept, and aiding recovery of imperilled communities and families. They map well onto the stepped strategic model of care that is policy in England and recommended by NATO.

The HPA found that there is a growing body of evidence which suggests that floods can have profound effects on people’s wellbeing, psychosocial resilience, relationships and mental health, often over extended periods of time. The lessons learned about the relationship between flooding and its impacts on mental health may be applicable to other disasters as well. *The effects of flooding on mental health: Outcomes and recommendations from a review of the literature* offers a summary of epidemiological research, government and non-government guidance on the effects of flooding on mental health and up-to-date strategies for mitigating the impacts of flooding on affected populations.
